# Rice flowering improves the muscle nutrient, intestinal microbiota diversity, and liver metabolism profiles of tilapia (*Oreochromis niloticus*) in rice-fish symbiosis

**DOI:** 10.1186/s40168-022-01433-6

**Published:** 2022-12-16

**Authors:** Erlong Wang, Ya Zhou, Yue Liang, Fei Ling, Xiaoshu Xue, Xianlin He, Xuliang Zhai, Yang Xue, Chunlong Zhou, Guo Tang, Gaoxue Wang

**Affiliations:** 1 College of Animal Science and Technology, Northwest A&F University, Yangling, 712100 Shaanxi China; 2 Northwest A&F University Shenzhen Research Institute, Shenzhen, 518000 Guangdong China; 3 Chongqing Three Gorges Vocational College, Chongqing, 404155 China; 4 Department of Chemical Engineering, Auburn University, Auburn, AL 36849 USA; 5 Chongqing Fisheries Technical Extension Center, Chongqing, 401121 China

**Keywords:** Rice-fish symbiosis, Tilapia, Muscle nutrient, Intestinal microbiota, Liver metabolomics

## Abstract

**Background:**

Rice-fish symbiosis, as an ecological and green aquaculture model, is an effective measure to relieve the environmental stress from intensive aquaculture. Compared with traditional aquaculture, the altered rearing pattern and environment will make differences in muscle nutrient and quality, intestinal microbiota, body metabolism, and even disease resistance in fish.

**Results:**

To investigate this, we explored the differences between rice-tilapia (aRT and bRT) and tank-tilapia (aTT and bTT) models at the periods before and after rice flowering using 16S rRNA sequencing and untargeted metabolomics. The results showed that compared with tilapia reared in the tank model, the fish body length and weight, the muscle total umami amino acid, and monounsaturated fatty acid content were obviously higher in the rice-fish model, especially after rice flowering. Compared with other groups, the intestinal microbiota diversity of fish in the bRT group was significantly higher; the dominant microbiota was *Bacteroidetes* and *Firmicutes* at the phylum level, *Bacteroides* and *Turicibacter* at the genus level, and the relative abundances of Gram-negative, potentially pathogenic, and stress-tolerant bacteria were the highest, lowest, and highest, respectively. Besides, the differential metabolite analysis indicated that rice-fish symbiosis improved the metabolic profiles and modulated the metabolic pathways in tilapia. Moreover, the correlation analysis of 16S sequencing and metabolomics showed that *Bacteroides* showed a positive correlation with many metabolites related to amino acid, fatty acid, and lipid metabolism.

Video Abstract

**Conclusions:**

In summary, rice flowering improves the tilapia muscle nutrient, intestinal microbiota diversity, and disease resistance and modulates the host metabolism to acclimatize the comprehensive environment in rice-fish symbiosis. Specifically, rice flowering alters the microbiota abundance involved in amino acid, fatty acid, and lipid metabolism, resulting in improving the muscle nutrient and quality through the crosstalk of gut microbial and host metabolism. Our study will provide not only new insight into the gut microbiota-metabolism-phenotype axis, but also strong support for the promotion and application of rice-fish symbiosis in aquaculture.

**Supplementary Information:**

The online version contains supplementary material available at 10.1186/s40168-022-01433-6.

## Background

In recent years, with the fast increase of aquaculture scales and quantities, intensive aquaculture has posed severe stress and challenges to the ecological environment. Compared with the traditional aquaculture model, rice-fish symbiosis, as an ecological and green planting and breeding model, is an effective measure to relieve the environmental stress from intensive aquaculture [1] and an integrated system to achieve production and environment sustainability [2]. Many practices have shown that the rice-fish coculture model possesses the advantages of “one water, dual use” and “one field, double harvesting” and can not only realize the rational utilization of resources and the maximization of economic benefits to obtain high-quality rice and fish, but also keep the environment from being polluted and damaged by the use of chemical fertilizers and pesticides in paddy fields and achieve the mutual benefit and win–win goal of rice and fish, benefit, and environment [3].

Tilapia (*Oreochromis niloticus*), as the second most cultured freshwater fish next to carp in the world [4], has played an important role in global aquaculture with many advantages, including strong disease resistance, fast growth, resistance to various environments, and capable of being produced in dense and ultra-dense forms [5, 6]. Therefore, tilapia has gained worldwide attention, and using new technologies and strategies to farm this species is crucially important for global aquaculture and agriculture. As a kind of omnivore species, tilapia can filter food particles and are easily fed with a rich natural food source. Moreover, tilapia usually takes 100–120 days to grow to the sale specification [7], which is synchronized with the rice growth cycle with the same temperature range [8]. Thus, tilapia is the ideal fish species for the symbiotic mode of rice and fish, which is also beneficial to control the weeds and insect pests in paddy fields. It has been reported that crab and crayfish in the corresponding rice-crab/rice-crayfish coculture model showed better growth performance and muscle quality than that of the monoculture system, with enhanced umami, sweetness, and overall flavor [9, 10]. The rice-fish coculture model could improve the economic performance of aquatic animals [11, 12].

However, at present, there was no systematic study to report the differences between rice-tilapia (aRT and bRT) and tank-tilapia (aTT and bTT) models on the muscle nutrient, intestinal microbiota diversity, and liver metabolism profiles at the periods before and after rice flowering. To investigate this, we compared the muscle amino acid and fatty acid content and employed 16S rRNA sequencing and untargeted metabolomics to identify the differential intestinal microbiota and liver metabolites and explored the correlation analysis of microbial and metabolism, which hope to provide novel insights into the theoretical foundation of rice-fish symbiosis.

## Materials and methods

### Experimental design and sampling

**Fig. 1:**
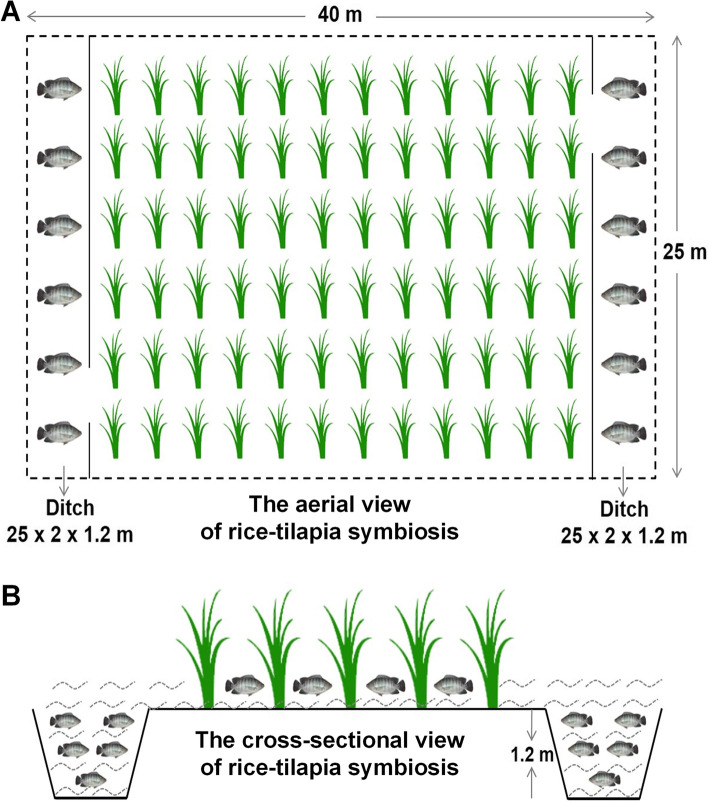
The aerial view and the cross-sectional view of the rice-fish symbiosis system. **A** The aerial view of the rice-fish symbiosis system. **B** The cross-sectional view of the rice-fish symbiosis system

The experiment was conducted at a specialized rice-fish coculture farm with many paddies (each measuring 40 m × 25 m) in Chongqing, China. The paddy had a two-side ditch design (25 m × 2.0 m × 1.2 m, length × width × depth), which provided appropriate space for rice cultivation and the refuge for tilapia reared in paddy (Fig. 1). Two fish tanks with 100 m^2^ were prepared as the control group. A total of 1200 healthy and similarly sized tilapia (17.24 ± 1.3 g) were selected and randomly put into two paddy fields and two fish tanks (each with 300 tails). The experiment was performed from April to August 2021, and the rice was transplanted on 20 April. We select 20 July as the date before rice flowering (named aRT in the paddy group and aTT in the tank group) and 20 August as the date after rice flowering (named bRT in the paddy group and bTT in the tank group) as shown in Table 1. Five fish were randomly sampled from each group, the body length and weight were measured, the muscle and liver were sampled with liquid nitrogen for testing, and the intestinal content was collected and stored at − 20 °C for testing.

**Table 1:** Experimental design and groups

Tilapia groups	Aquaculture models	Sampling time
aRT	Rice-tilapia (paddy group)	20 July
bRT	Rice-tilapia (paddy group)	20 August
aTT	Tank-tilapia (tank group)	20 July
bTT	Tank-tilapia (tank group)	20 August

### Amino acid and fatty acid contents in muscle

The muscle nutrient was evaluated by detecting the amino acid composition and fatty acid content according to previous studies [13]. Briefly, the muscle sample was ground into powder with liquid nitrogen; 40 mg freeze-dried muscle powder and 10 mL of 6 M hydrochloric acid were placed in a 50-mL ampoule, which was then placed in a constant temperature drying oven to hydrolyze for 24 h. The hydrolysate was diluted with deionized water, transferred to the rotary evaporator flask, and evaporated to dryness (60 °C). The evaporating product was washed with 0.02 M hydrochloric acid and transferred to another volumetric flask; 3-mL hydrolysate sample and standard amino acid solution were respectively filtered into an automated sample injection bottle, the amino acid content was determined by the automatic amino acid analyzer (LA8080, Hitachi, Japan).

The fatty acid content was determined according to the following steps: 0.5 g freeze-dried muscle powder and a 4-mL mixture of benzene and petroleum ether (volume ratio 1:1) were put into a 10-mL centrifuge tube to extract for 24 h; 4-mL potassium hydroxide-methanol solution (0.4 M) was added into the centrifuge tube for methyl esterification, and the mixture was vortexed for 3 min and stood for 30 min. The mixture was diluted with deionized water, and the upper layer solution was extracted. Two hundred microliters of upper-layer solution and 800 μL hexane were mixed and filtered with a 0.22-μm filter membrane. The gas chromatograph GC 2010 (A09056) was used to determine the fatty acid content.

### 16 s rRNA gene sequencing for the intestinal microbiota analysis

The genomic DNA of different samples were extracted according to the E.Z.N.A. ®Stool DNA Kit (D4015, Omega, Inc., USA). The total DNA was eluted in 50 μL elution buffer and stored at − 80 °C until use in the PCR by LC-Bio Technology (Hangzhou, China). PCR amplification was performed in a 25-μL reaction mixture to obtain the V3–V4 regions of 16 s rRNA gene using the primers 341F (5′-CCTACGGGNGGCWGCAG-3′) and 806R (5′-GGACTACHVGGGTATCTAATCC-3′). The 5′ ends of the primers were tagged with specific barcodes for each sample and were sequenced with universal primers. The PCR condition consisted of an initial denaturation at 98 ℃ for 30 s; 32 cycles of denaturation at 98 ℃ for 10 s, annealing at 54℃ for 30 s, and extension at 72 ℃ for 45 s; and then final extension at 72 ℃ for 10 min. The PCR product was confirmed with 2% agarose gel electrophoresis, purified by AMPure XT beads (Beckman Coulter Genomics, Danvers, USA), and quantified by Qubit (Invitrogen, USA). The amplicon pools were prepared for sequencing, and the size and quantity of the amplicon library were assessed using Agilent 2100 Bioanalyzer (Agilent, USA) and Library Quantification Kit for Illumina (Kapa Biosciences, Woburn, USA), respectively. The libraries were sequenced on the NovaSeq PE250 platform for generating 250-bp paired-end reads in sequence.

### LC–MS conditions for metabolomics analysis

The metabolomics analysis of the liver sample was conducted using an ultra-performance liquid chromatography (UPLC) system (SCIEX, UK). An Acquity UPLC T3 column (100 mm × 2.1 mm, 1.8 µm, Waters, UK) was used for the reversed-phase separation and maintained at 35 ℃. The mobile phase consisted of solvent A (water with 0.1% formic acid), and solvent B (acetonitrile with 0.1% formic acid) was introduced for the metabolite separation. The gradient elution conditions were as follows with a flow rate of 0.4 mL/min: 5% solvent B for 0–0.5 min, 5–100% solvent B for 0.5–7.0 min, 100% solvent B for 7.0–8.0 min, 100–5% solvent B for 8.0–8.1 min, and 5% solvent B for 8.1–10.0 min.

The TripleTOF 5600 Plus high-resolution tandem mass spectrometer (SCIEX, Warrington, UK) was used to detect the eluted metabolites. The Q-TOF was operated in both positive and negative ion modes. The ion spray floating voltage was set at 5 kV under positive ion mode and − 4.5 kV under negative ion mode. The mass spectrometry (MS) data was acquired in the IDA mode. The TOF mass range was from 60 to 1200 Da. The survey scans were acquired in 150 ms, and as many as 12 product ion scans were collected if they exceeded a threshold of 100 counts per second (counts/s) with a 1 + charge state. The total cycle time was fixed to 0.56 s. Four-time bins were summed for each scan at a pulse frequency of 11 kHz by monitoring the 40-GHz multichannel TDC detector with four‐anode/channel detection. Dynamic exclusion was set as 4 s. During the entire acquisition period, the mass accuracy was calibrated after every 20 samples. Furthermore, a quality control (QC) sample was analyzed after every 10 samples to evaluate the stability of LC–MS during the whole acquisition.

### Data analysis

#### 16S rRNA data analysis

Paired-end reads were assigned to samples based on their unique barcode and truncated by cutting off the barcode and primer sequence, then merged using FLASH. Quality filtering on the raw reads was performed under specific filtering conditions to obtain high-quality clean tags according to the fqtrim (v0.94). Chimeric sequences were filtered using the Vsearch software (v2.3.4). The feature table and sequence were obtained after dereplication with DADA2. According to SILVA (release 132) classifier, feature abundance was normalized using the relative abundance of each sample. The α-diversity was analyzed to evaluate the species complexity through 5 indices, including observed species, Chao1, Shannon, Simpson, and Good’s coverage using QIIME2. The β-diversity was analyzed to investigate the differences within and between groups by the principal component analysis (PCA), principal coordinates analysis (PCoA), and UPGMA clustering analysis. Blast was used for sequence alignment, and the feature sequences were annotated with the SILVA database for each representative sequence. Besides, the microbial species and potential phenotypes were also identified and analyzed with QIIME2. All the data processing and analysis were performed using the OmicStudio tools at https://www.omicstudio.cn/tool.

#### Metabolomics data processing

The acquired LC–MS data pretreatments including peak picking, peak grouping, retention time correction, second peak grouping, and annotation of isotopes and adducts were performed using the XCMS software. The raw data files were converted into mzXML format and then processed using the XCMS, CAMERA, and metaX toolbox included in the R software (AT&T, USA). Each ion was identified by combining retention time (RT) and m/z data. The intensity of each peak was recorded, and a three-dimensional matrix containing arbitrarily assigned peak indices (retention time–m/z pairs), sample names (observations), and ion intensity information (variables) was generated. The metabolites were annotated by matching the exact molecular mass data (m/z) of samples with those from the online databases, including the Kyoto Encyclopedia of Genes and Genomes (KEGG) and Human Metabolome Database (HMDB). If the mass difference between the observed value and the database value was < 10 ppm, the metabolite would be annotated and the molecular formula of the metabolite would be identified and validated by the isotopic distribution measurements. We also used an in-house fragment spectrum library of metabolites to validate the metabolite identification.

The intensity of the peak data was further pre-processed by metaX. Features that were detected in < 50% of the QC samples or 80% of the biological samples were removed, and the remaining peaks with missing values were imputed with the *k*-nearest neighbor algorithm to further improve the data quality. PCA was performed to detect the outlier and evaluate the batch effects using the pre-processed dataset. QC-based robust LOESS signal correction was fitted to the QC data with respect to the order of injection to minimize signal intensity drift over time. In addition, the relative standard deviations (SD) of the metabolic features were calculated across all QC samples, and those > 30% were then removed. The analysis methods included PCA and PLS-DA. The MetaX software was used to quantify differential metabolites and differential metabolite screenings. Supervised PLS-DA was conducted using metaX to discriminate the different variables between the groups. The variable importance in projection (VIP) was calculated, and a VIP cutoff of 1.0 was used to select important features (VIP ≥ 1; ratio ≥ 2 or ratio ≤ 1/2; *q* ≤ 0.05). The differential metabolites and metabolic pathways were also explored through pairwise comparison. The corresponding graphs and heatmaps were drawn using the OmicStudio tools at https://www.omicstudio.cn/tool.

### Statistical analysis

One-way analysis of variance (ANOVA) was used for evaluating the between-group significant differences using SPSS 25.0 (Chicago, IL, USA). *p* < 0.05 was set as the significance threshold. Spearman’s correlation analysis was conducted to analyze the correlation relationship between the microbiota and metabolite using the data of significantly differential genus-level microbiota and differential secondary metabolites. A scaled heatmap was constructed for the correlation matrix using the default clustering method.

## Results

### Tilapia growth performance

**Fig. 2:**
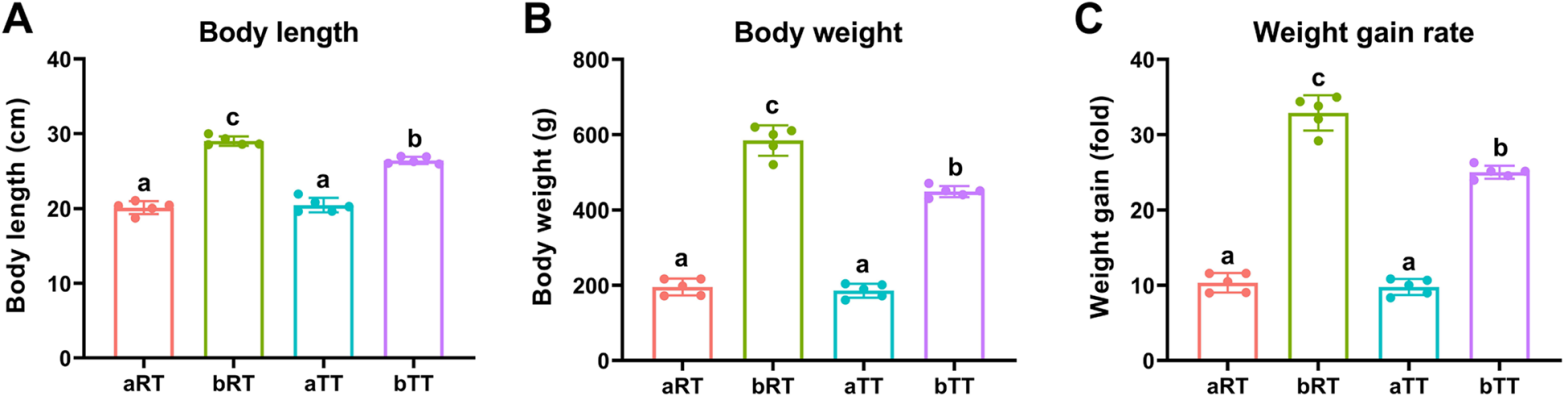
The body length, weight, and weight gain rates of tilapia in each group. **A** Body length. **B** Body weight. **C** Weight gain rates. Different letters represent significant differences observed between various groups (*p* < 0.05). Weight gain rate = (*W*_final_ − *W*_initial_)/*W*_initial_

The results indicated that before rice flowering, the body length of tilapia in the paddy group (aRT) was a little shorter (*p* > 0.05) than that in the tank group (aTT). In contrast, after rice flowering, the body length of tilapia in the paddy group (bRT) was significantly (*p* < 0.05) longer than that in the tank group (bTT). Besides, the body weight and weight gain of tilapia in the paddy group were higher than that in the tank group at the same time, especially after rice flowering (Fig. 2).

### Amino acid and fatty acid content in muscle

The results indicated that there was no significant difference in single amino acid content in the four groups. However, as shown in Table 2, the total essential amino acid (TEAA), total amino acid (TAA), and total umami amino acid (TUAA) in the bRT group were significantly (*p* < 0.05) higher than that in the aRT group and little higher than that in the aTT and bTT groups, while the total non-essential amino acid (TNEAA) content in four groups was in the order of bTT > bRT > aTT > aRT. Besides, the values of TUAA/TAA (%) in paddy groups (aRT and bRT) were both higher than that in tank groups (aTT and bTT) at the same time with the order of bRT > bTT > aRT > aTT, while the values of TEAA/TAA (%) and TEAA/TNEAA (%) in the four groups were both in the order of bRT > aTT > bTT > aRT.

**Table 2:** The amino acid content in tilapia muscle

Amino acids (AA)	aRT	bRT	aTT	bTT
Thr^a^	3.52 ± 0.18	3.71 ± 0.07	3.64 ± 0.13	3.70 ± 0.12
Val^a^	3.76 ± 0.20	4.01 ± 0.11	3.94 ± 0.15	3.93 ± 0.15
Met^a^	2.21 ± 0.15	2.40 ± 0.04	2.35 ± 0.08	2.41 ± 0.08
Ile^a^	3.46 ± 0.21	3.76 ± 0.07	3.68 ± 0.14	3.71 ± 0.13
Leu^a^	6.45 ± 0.37	6.90 ± 0.14	6.81 ± 0.25	6.87 ± 0.22
Lys^a^	7.29 ± 0.39	7.81 ± 0.16	7.69 ± 0.29	7.73 ± 0.25
Phe^a,b^	3.39 ± 0.17	3.57 ± 0.07	3.53 ± 0.12	3.53 ± 0.11
Asp ^b^	7.68 ± 0.49	8.31 ± 0.22	8.27 ± 0.29	8.28 ± 0.25
Glu^b^	13.20 ± 0.85	14.16 ± 0.22	13.98 ± 0.39	14.28 ± 0.40
Gly^b^	4.43 ± 0.17	4.35 ± 0.11	3.95 ± 0.02	4.29 ± 0.41
Ala^b^	4.93 ± 0.24	5.06 ± 0.07	5.07 ± 0.12	5.05 ± 0.18
Tyr^b^	2.17 ± 0.12	2.32 ± 0.03	2.32 ± 0.12	2.31 ± 0.06
Arg	4.84 ± 0.19	5.04 ± 0.06	5.03 ± 0.14	5.19 ± 0.22
Cys	0.67 ± 0.06	0.71 ± 0.02	0.66 ± 0.04	0.68 ± 0.06
His	2.29 ± 0.10	2.31 ± 0.07	2.42 ± 0.08	2.34 ± 0.11
Ser	3.14 ± 0.14	3.21 ± 0.06	3.21 ± 0.10	3.25 ± 0.10
Pro	2.69 ± 0.11	2.48 ± 0.03	2.52 ± 0.06	2.5 ± 0.19
TEAA	30.08 ± 1.66^a^	32.15 ± 0.65^b^	31.64 ± 1.15^ab^	31.88 ± 1.05^b^
TNEAA	46.05 ± 2.31^a^	47.94 ± 0.80^b^	47.44 ± 1.12^ab^	48.19 ± 1.75^b^
TUAA	35.81 ± 1.96^a^	37.76 ± 0.66^b^	37.12 ± 1.01^ab^	37.73 ± 1.21^b^
TAA	76.13 ± 3.94^a^	80.10 ± 1.45^b^	79.08 ± 2.25^b^	80.07 ± 2.75^b^
TEAA/TAA (%)	39.51 ± 0.36	40.14 ± 0.10	40.01 ± 0.31	39.82 ± 0.32
TUAA/TAA (%)	47.04 ± 0.29	47.14 ± 0.05	46.94 ± 0.06	47.11 ± 0.12
TEAA/TNEAA (%)	65.32 ± 0.97^a^	67.06 ± 0.28^b^	66.69 ± 0.85^ab^	66.15 ± 0.90^ab^

Different letters in the same row represent the significant differences observed between various groups (*p* < 0.05)

*TEAA* total essential amino acid, *TNEAA* total non-essential amino acid, *TAA* total amino acid, *TUAA* total umami amino acid

^a^Essential amino acid

^b^Umami amino acid

The results of fatty acid content suggested that the differences in saturated fatty acid (SFA) content in the four groups were not significant (Table 3). The content of monounsaturated fatty acids (MUFA) in paddy groups (aRT and bRT) were both higher than that in tank groups (aTT and bTT) at the same time with the order of bRT > aRT > aTT > bTT, while the polyunsaturated fatty acid (PUFA) content in paddy groups (aRT and bRT) were both lower than that in tank groups (aTT and bTT) at the same time with the order of bTT > aTT > aRT > bRT.

**Table 3:** The fatty acid content in tilapia muscle

Fatty acid (%)	aRT	bRT	aTT	bTT
Myristic acid (C14:0)	1.36 ± 0.07	1.61 ± 0.21	1.55 ± 0.14	1.56 ± 0.19
Palmitic acid (C16:0)	20.07 ± 1.06	20.97 ± 0.67	20.50 ± 0.36	20.47 ± 0.90
Stearic acid (C18:0)	5.58 ± 0.48	6.26 ± 0.31	6.72 ± 0.13	6.41 ± 0.04
Arachidic acid (C20:0)	0.20 ± 0.04	0.24 ± 0.01	0.08 ± 0.14	0.16 ± 0.14
SFA	27.20 ± 1.52	29.08 ± 0.41	28.84 ± 0.57	28.59 ± 1.20
Palmitoleic acid (C16:1)	2.95 ± 0.22	2.99 ± 0.27	2.47 ± 0.13	2.07 ± 0.42
Oleic acid (C18:1)	30.33 ± 2.90^a^	30.17 ± 0.29^a^	28.23 ± 0.86^ab^	27.67 ± 1.32^b^
Eicosenoic acid (C20:1)	1.27 ± 0.13	1.40 ± 0.02	1.45 ± 0.12	1.31 ± 0.08
MUFA	34.55 ± 3.12^a^	34.56 ± 0.33^a^	32.16 ± 0.94^b^	31.04 ± 1.41^b^
Linoleic acid (C18:2)	21.17 ± 2.82^a^	21.20 ± 0.70^ab^	23.43 ± 0.91^bc^	25.17 ± 1.96^c^
Linolenic acid (C18:3n-3)	2.12 ± 0.24	1.99 ± 0.11	2.14 ± 0.05	2.22 ± 0.21
Docosahexaenoic acid (C22:6)	2.11 ± 0.16	1.05 ± 0.06	1.42 ± 0.49	0.91 ± 0.07
PUFA	25.40 ± 2.88^ab^	24.25 ± 0.73^a^	26.99 ± 0.47^bc^	28.30 ± 2.04^c^
Other fatty acids	12.70 ± 1.80	12.03 ± 0.29	12.10 ± 1.13	12.10 ± 0.70

Different letters in the same row represent the significant differences observed between various groups (*p* < 0.05)

*SFA* Saturated fatty acid, *MUFA* Monounsaturated fatty acid, *PUFA* Polyunsaturated fatty acid

### Intestinal microbiota characteristics

#### Operational taxonomic unit (OTU) analysis

**Fig. 3:**
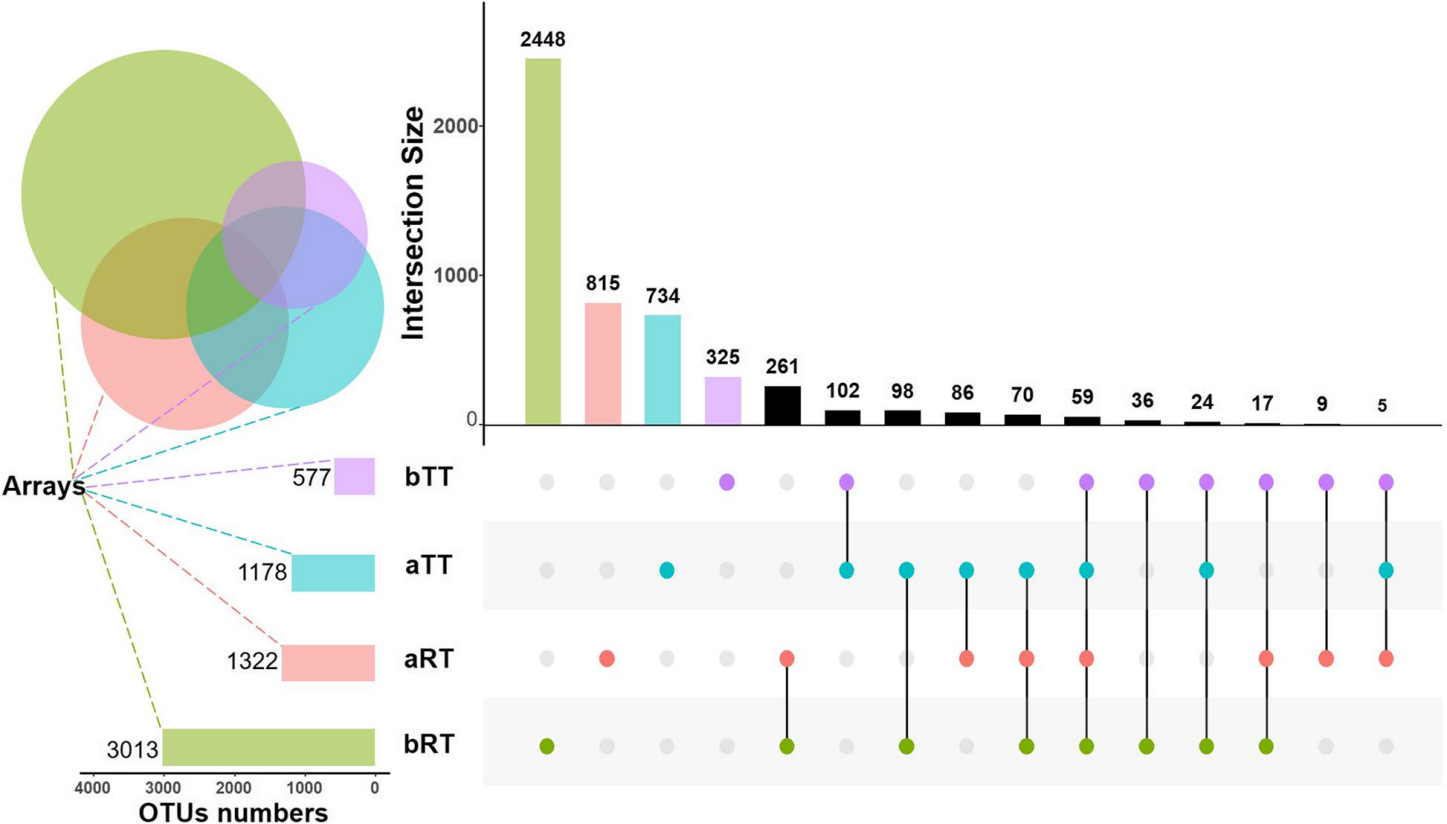
The Venn diagram and OTU numbers of intestinal microbiota in the four groups

The OTU analysis indicated that the total number of OTUs in four groups was 5089. The paddy model and tank model shared 59 OTUs, the bRT and aRT groups shared 407 OTUs, and the bRT and bTT groups shared 136 OTUs. However, at the same time, the total OTU number in the paddy model was higher than that in the tank model. The total OTU number in the bRT group was highest (3013) with 2448 specific OTUs (Fig. 3).

#### Intestinal microbiota diversity analysis

**Fig. 4:**
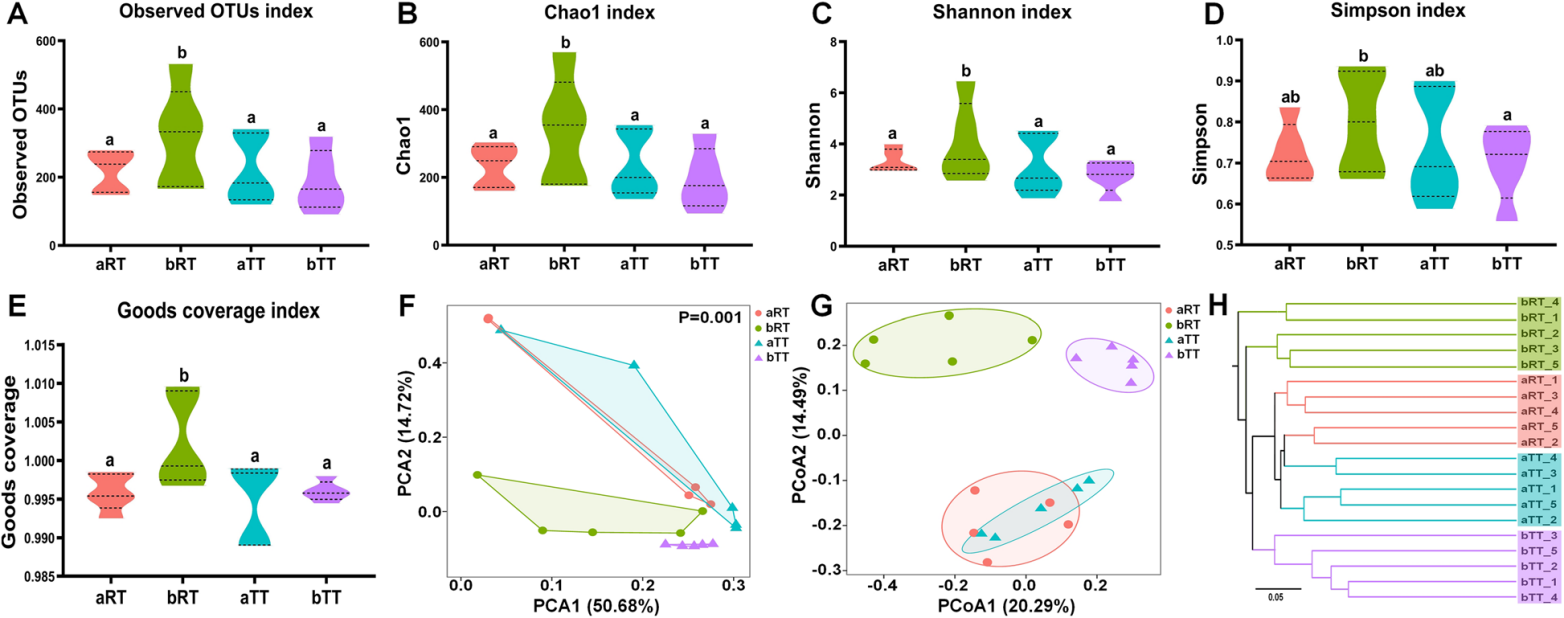
The α and β diversity analyses of intestinal microbiota in the four groups. **A** The observed OTU index. **B** Chao1 index. **C** Shannon index. **D** Simpson index. **E** Goods coverage index. **F** The PCA analysis. **G** The PCoA analysis. **H** The UPGMA cluster analysis. Different letters above the columns represent the significant differences observed between various groups (*p* < 0.05)

According to the microbiota α-diversity analysis, the observed OTUs, chao1, Shannon, and goods coverage indexes in the bRT group were all significantly (*p* < 0.05) higher than those in the other three groups (Fig. 4), and the Simpson index in the bRT group was significantly (*p* < 0.05) higher than that in the bTT group and slightly higher (*p* > 0.05) than those in aRT and aTT groups. There was no significant difference among the aRT, aTT, and bTT groups. As for the β-diversity, the results of PCA and PCoA analyses both indicated that the samples of the bRT and bTT groups clustered better than that of the aRT and aTT groups, especially the bTT group. The UPGMA hierarchical clustering analysis showed that five samples in each group were all clustered together.

**Fig. 5:**
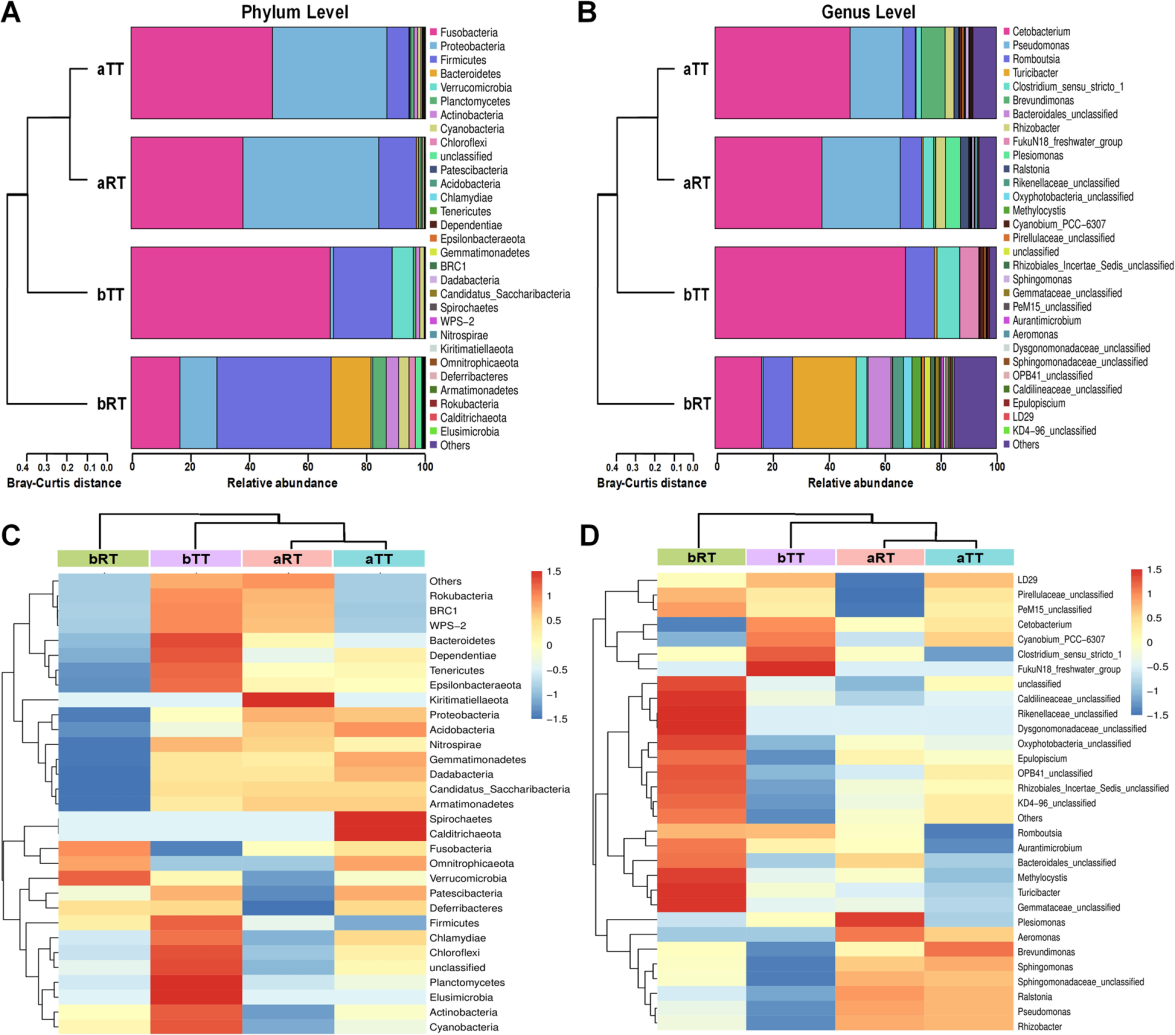
The composition and clustering analysis of intestinal microbiota at phylum and genus levels in the four groups. **A** The composition analysis of intestinal microbiota at the phylum level. **B** The composition analysis of intestinal microbiota at the genus level. **C** The heat map and clustering analysis of intestinal microbiota at the phylum level. **D** The heat map and clustering analysis of intestinal microbiota at the genus level

#### Microbial species analysis

**Fig. 6:**
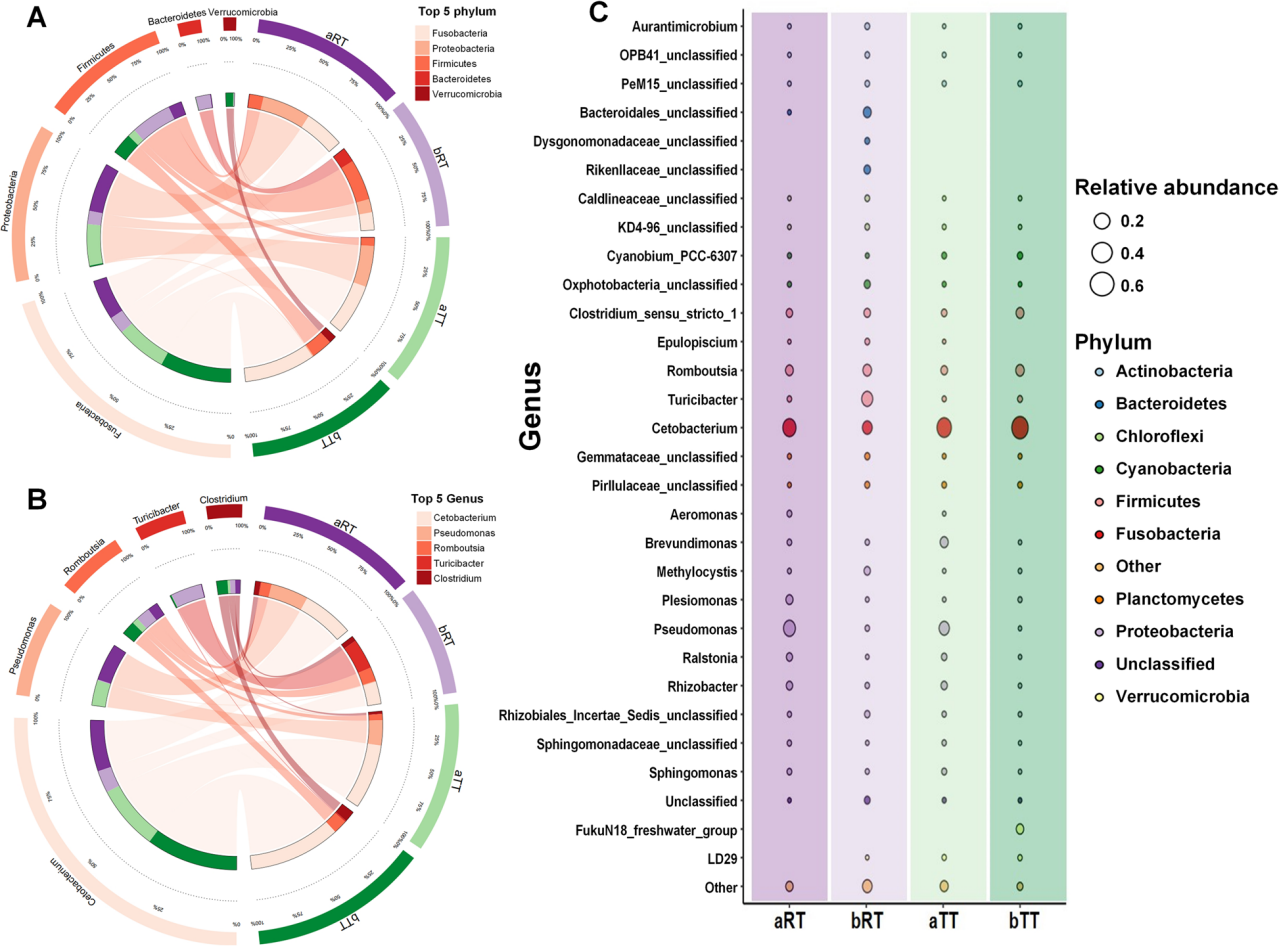
The proportions of the top 5 phyla and genera and the relative abundance (bubble plot) in the four groups. **A** The top 5 phyla of intestinal microbiota in the four groups. **B** The top 5 genera of intestinal microbiota in the four groups. **C** The relative genus abundance (bubble plot) of intestinal microbiota in the four groups

The results showed that, at the phylum level, *Fusobacteria*, *Proteobacteria*, *Firmicutes*, *Bacteroidetes*, and *Verrucomicrobia* were the top 5 most microbes in the four groups (Figs. 5A, C and 6A). The most microbes in the aRT, bRT, aTT, and bTT groups were *Proteobacteria* (46.16%), *Firmicutes* (37.78%), *Fusobacteria* (48.04%), and *Fusobacteria* (67.69%), respectively. No matter in the paddy model or tank model, the top 2 most microbes before rice flowering were both *Fusobacteria* and *Proteobacteria*, and after rice flowering, both *Fusobacteria* and *Firmicutes*. Moreover, the *Bacteroidetes* proportion in the bRT group was significantly higher than the other groups. Besides, at the genus level, the top 5 microbes in the four groups were *Cetobacterium*, *Pseudomonas*, *Romboutsia*, *Turicibacter*, and *Clostridium_sensu_stricto_1* (Figs. 5B, D and 6B). The most microbes in the aRT, bRT, aTT, and bTT groups were *Cetobacterium* (38.01%), *Turicibacter* (22.62%), *Cetobacterium* (48.03%), and *Cetobacterium* (67.68%), respectively. Before rice flowering, the top 2 most microbes were both *Cetobacterium* and *Pseudomonas*. While after rice flowering, the top 2 were *Turicibacter* and *Cetobacterium* in the bRT group and *Cetobacterium* and *Romboutsia* in the bTT group. Similar to the phylum level, the *Bacteroides* proportion in the bRT group was higher than in the other groups. In addition, the results of relative abundance (Fig. 6C) showed that *Dysgonomonadaceae*_*unclassified* and *Rikenellaceae*_*unclassified* only existed in the bRT group, *FukuN18*_*freshwater*_*group* only appeared in the bTT group, *Bacteroidales*_*unclassified* only appeared in the paddy model, and *Aeromonas* genus only existed at the period before rice flowering.

#### Microbial potential phenotype analysis

**Fig. 7:**
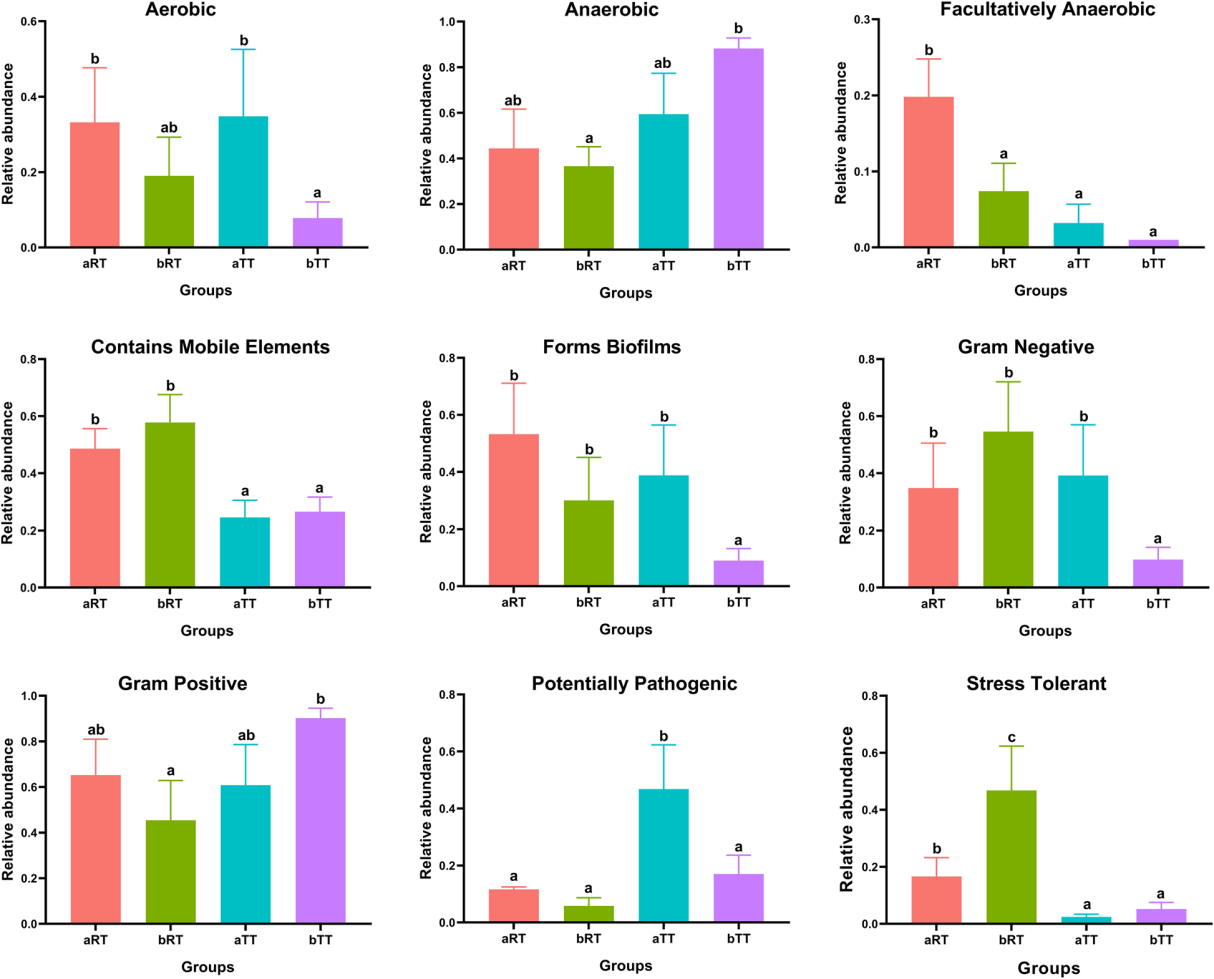
The nine potential phenotype analyses of intestinal microbiota in the four groups. Different letters above the columns represent the significant differences observed between various groups (*p* < 0.05)

Furthermore, to better understand the relationship between the intestinal microbiota composition and host health, nine potential bacteria phenotypes, including aerobic, anaerobic, facultatively anaerobic, contains mobile elements, forms biofilms, Gram-negative, Gram-positive, potentially pathogenic, and stress-tolerant bacteria, were predicted and analyzed in the four groups (Fig. 7). The results showed that compared with the period after rice flowering, the relative abundances of aerobic, facultatively anaerobic, forms biofilms, and potentially pathogenic bacteria of fish at the period before rice flowering were higher. Compared with the other three groups, the relative abundances containing mobile elements and Gram-negative bacteria in the bRT group were highest, and the relative abundances of Gram-positive and potentially pathogenic bacteria were lowest. Especially, the relative abundance of stress-tolerant bacteria in the bRT group was significantly (*p* < 0.05) higher than the other three groups.

### Metabolomics analysis

**Fig. 8:**
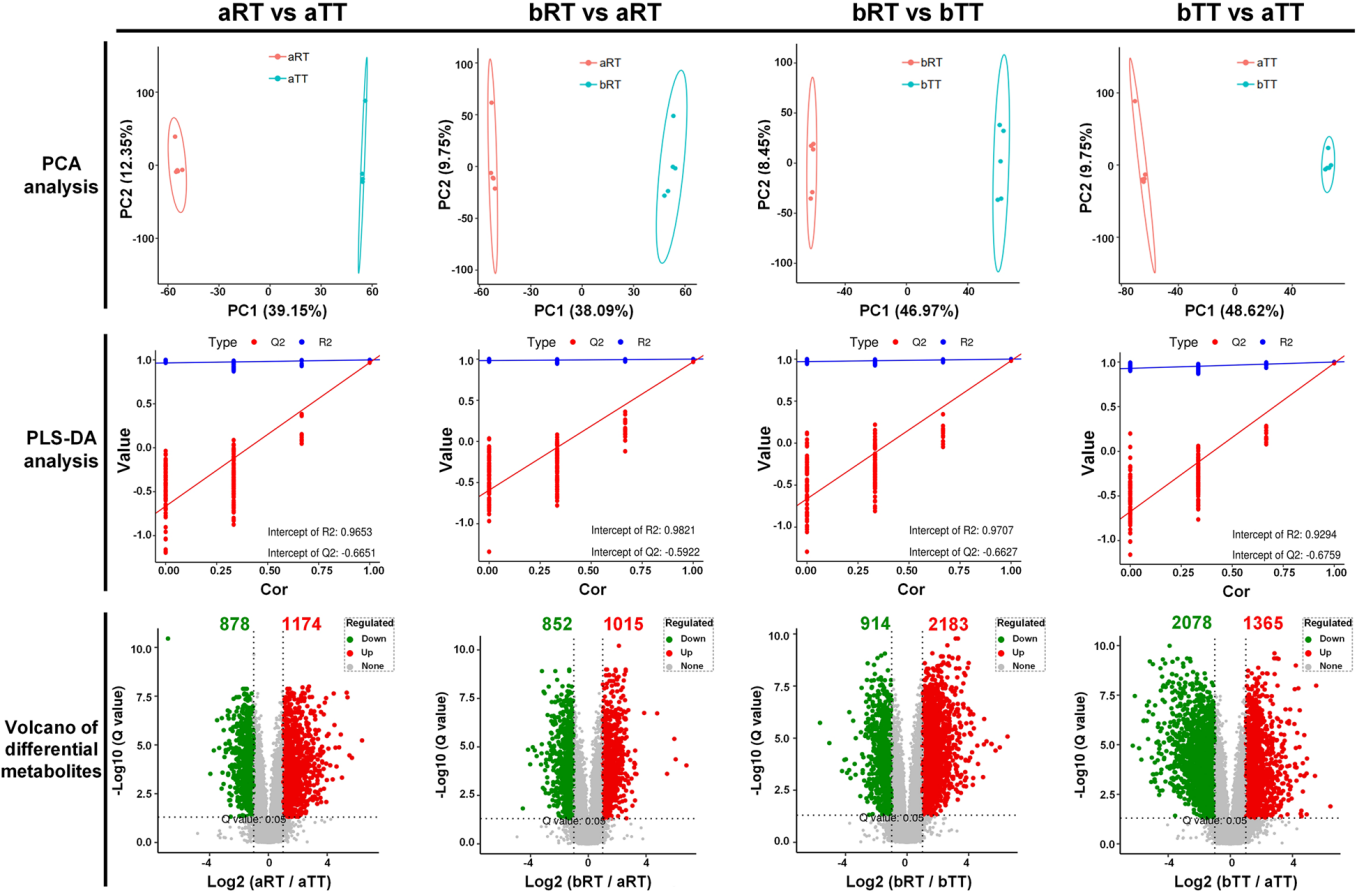
The PCA, PLS-DA, and differential metabolite analysis in the pairwise comparison including aRT vs aTT, bRT vs aRT, bRT vs bTT, and bTT vs aTT

The results of PCA analysis showed that there was no overlap between aRT and aTT, bRT and aRT, bRT and bTT, and bTT and aTT (Fig. 8). The results of PLS-DA analysis indicated that the Q2 values were all less than 0, suggesting significant differences in liver metabolites among the four groups (*p* < 0.05), and the differences between the various groups were higher than that between samples within the same group. Besides, compared with the aTT group, there were 878 downregulated and 1174 upregulated metabolites in the aRT group. Compared with the aRT group, the number of downregulated and upregulated metabolites in the bRT group was 852 and 1015, respectively. Compared with the bTT group, there were 914 downregulated and 2183 upregulated metabolites in the bRT group. Compared with the aTT group, the number of downregulated and upregulated metabolites in the bTT group was 2078 and 1365, respectively.

**Fig. 9:**
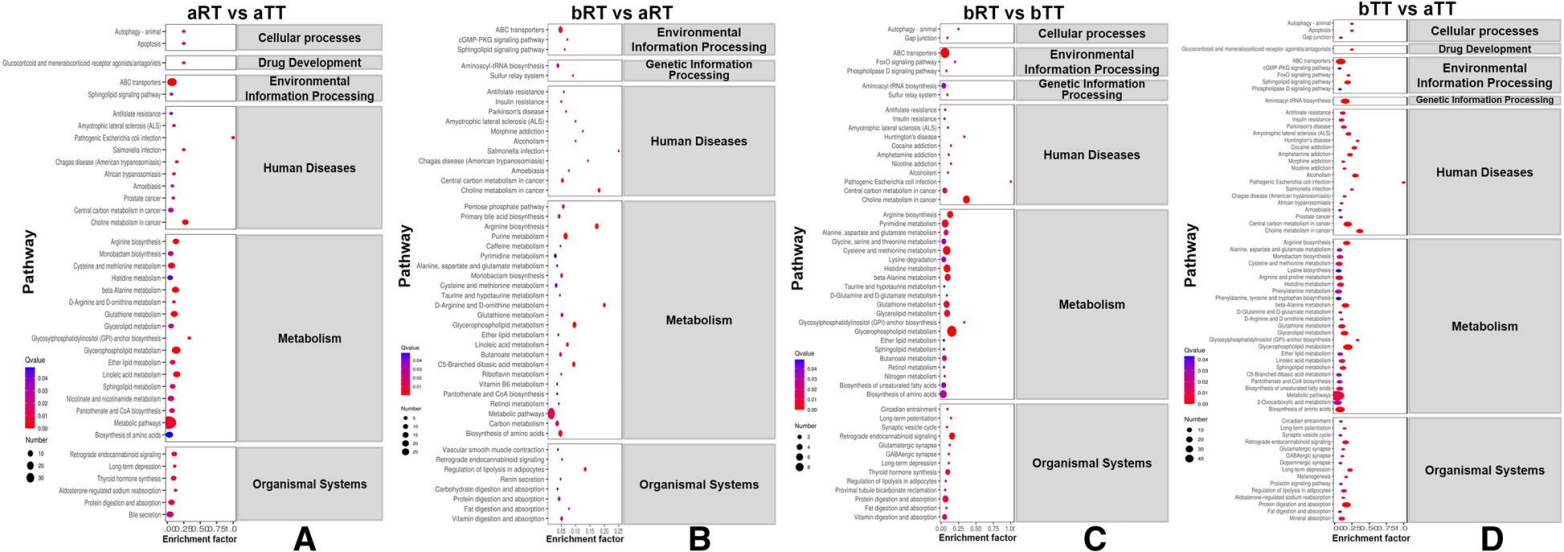
The pairwise comparison of differential metabolic pathways in the four groups. **A** aRT vs aTT. **B** bRT vs aRT. **C** bRT vs bTT. **D** bTT vs aTT

The results of the differential metabolic pathways indicated that, in aRT vs aTT, the ABC transporters, glycerophospholipid metabolism, and metabolic pathways in the aRT group were mainly upregulated, which were related to the environmental information processing and metabolism activities (Fig. 9A). However, compared with the aRT group, the bRT group upregulated the ABC transporters, purine metabolism, glycerophospholipid metabolism, and metabolic pathways (Fig. 9B). In bRT vs bTT, the ABC transporters, choline, pyrimidine, and glycerophospholipid metabolism in the bRT group were mainly upregulated, which were related to the environmental information processing, host diseases, and metabolism activities (Fig. 9C). Compared with the aTT group, the bTT group upregulated the ABC transporters, aminoacyl-tRNA biosynthesis, and metabolic pathways, which were related to the environmental information, genetic information processing, and metabolism activities (Fig. 9D).

### Correlation analysis of 16S sequencing and metabolomics

**Fig. 10:**
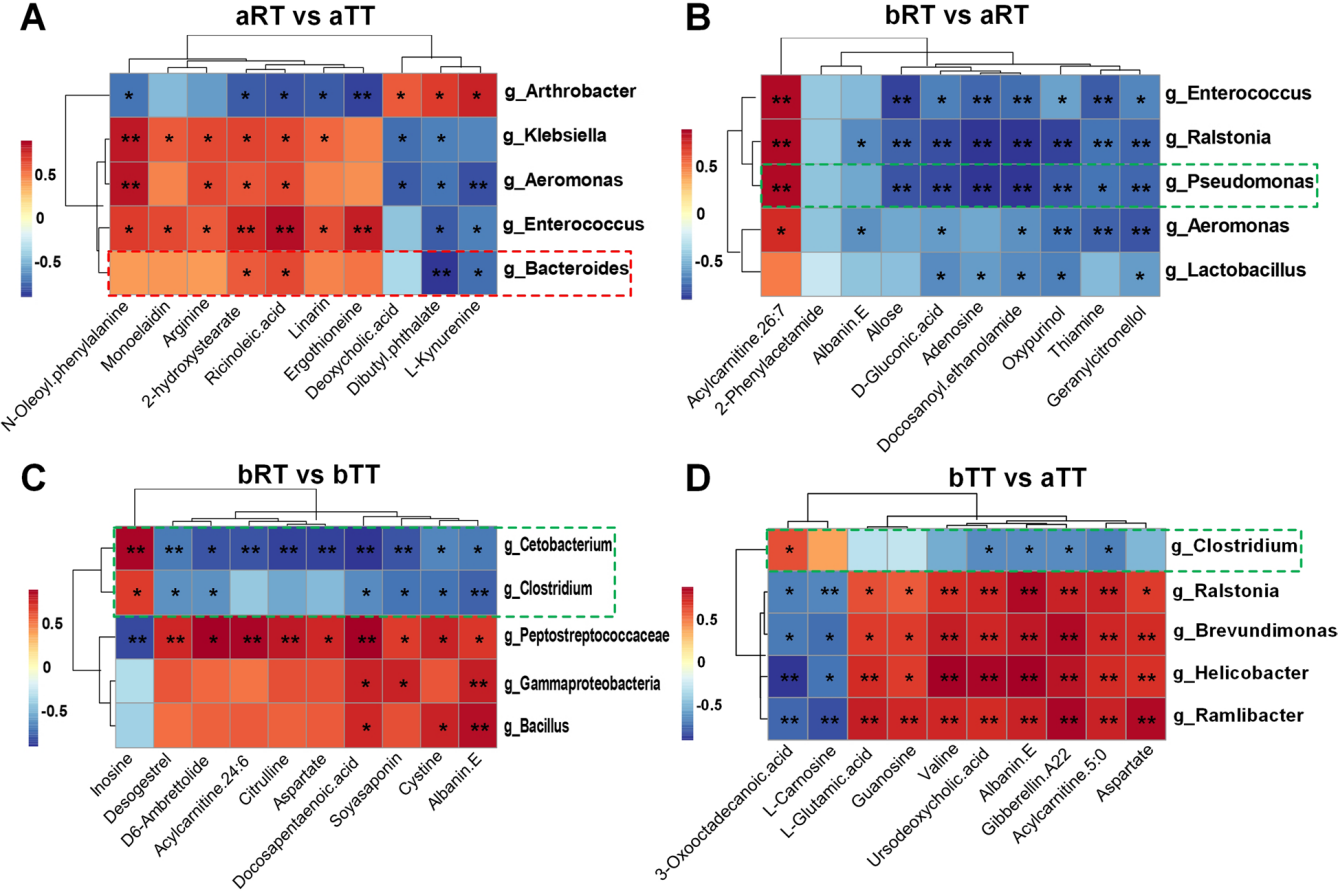
The combination analysis of differential flora and differential metabolites in pairwise comparison of the four groups. **A** aRT vs aTT. **B** bRT vs aRT. **C** bRT vs bTT. **D** bTT vs aTT. *p < 0.05, **p < 0.01

The correlation analysis of 16S sequencing and metabolomics was performed to explore the relationship between the intestinal microbiota and host metabolism using the data of differential genus-level microbiota and differential secondary metabolites by Spearman’s correlation analysis (Fig. 10). According to the genus-level microbial species analysis, compared with the other three groups, the relative abundance of *Bacteroides* in the bRT group was higher, while the relative abundance of *Pseudomona*, *Cetobacterium*, and *Clostridium* were lower. Spearman’s correlation analysis showed that *Bacteroides* was positively correlated with arginine, *N*-oleoyl phenylalanine, monoelaidin, 2-hydroxystearate, ricinoleic acid, linarin, and ergothioneine (Fig. 10A). In bRT vs aRT, five genera including *Pseudomona* showed negative correlation with all metabolites except acylcarnitine 26:7 (Fig. 10B). In bRT vs bTT, *Cetobacterium* and *Clostridium* showed negative correlation with all metabolites except inosine (Fig. 10C). Besides, in bTT vs aTT, *Clostridium* also showed negative correlation with eight metabolites including aspartate, valine, and l-glutamic acid (Fig. 10D).

## Discussion

As an ecological and green aquaculture model, rice-fish symbiosis has been designated a “globally important agricultural heritage system” [1] and exhibits many advantages, including improving the rice and fish quality, increasing the comprehensive benefits of paddy fields, and achieving the sustainable development of aquaculture industry and environment compared with traditional aquaculture. However, different rearing patterns and environments will make differences in growth performance, muscle quality and nutrients, intestinal microbiota, body metabolism, and even disease resistance in fish.

### Rice flowering improves the growth performance and muscle quality of tilapia in rice-fish symbiosis

Our study revealed that compared with the traditional tank-reared fish, the body weight and weight gain rate of tilapia in the paddy groups were obviously higher, especially during the period after rice flowering (i.e., bRT vs bTT), indicating that the rice-fish coculture and the fallen pollen after rice flowering may be helpful to improve the tilapia growth performance, which was similar with the results in rice-crab and rice-crayfish coculture systems [9, 10]. Besides, the muscle TUAA proportion of tilapia in the paddy groups (aRT and bRT) was both higher than that in the tank groups at the same time, which was consistent with the findings in rice-fish [14] and rice-shrimp [15] coculture systems that the paddy model improves the overall fish umami than tank model. Moreover, the flavor and tenderness of fish meat will be affected by the muscle fatty acid (SFA and MUFA) content [13]. In this study, the SFA and MUFA proportions in bRT were higher than those of the bTT group, especially MUFA, which indicated that rice-fish coculture, especially after rice flowering, could improve the fish muscle quality.

### Rice flowering increases the intestinal microbiota diversity and disease resistance of tilapia in rice-fish symbiosis

The intestinal microbiota is one of the key factors regulating intestinal immunity, nutrient absorption, and host healthy status [16–19]. However, its composition and diversity were reported to be affected by the culture environment, including the water salinity and pH, feed source, and season [20–24]. In this study, the OTU number of intestinal microbiota in paddy groups was much higher than that in the tank groups at the same time, and the α-diversity in bRT (after rice flowering) was significantly higher than that of the bTT group. Moreover, in combination with the β diversity analysis, it was speculated that the rice-fish coculture could enhance the species abundance and diversity of tilapia intestinal microbiota. It had been reported that the *Firmicutes* and *Bacteroidetes* were the predominant phyla colonizing the healthy gut and played essential roles in host health-promoting, immunity, and homeostasis [25, 26]. Our results showed that compared with the other groups, the relative abundance of *Firmicutes*, *Bacteroidetes*, and *Bacteroides* of fish in the bRT group were all the highest, which may be related with that tilapia feed on the fallen rice pollen and natural food after rice flowering in rice-fish coculture. In addition, the relative abundances of Gram-negative, potentially pathogenic, and stress-tolerant bacteria in the bRT group were the highest, lowest, and highest, respectively, which was consistent with the discovery that the relative abundance of Gram-negative bacteria can reflect the fish health [27, 28]. Taken together, it could be deduced that the fallen pollen after rice flowering could improve disease resistance and immunity in tilapia, since the flower pollen had been reported as fish diets with high levels of amino acids, fatty acids, and lipids [29, 30] and promoted the growth, improved the muscle quality, and enhanced the host stress tolerance and immunity in common carp [30], rainbow trout [31], and milkfish [32], while the detailed underlying mechanism needs to be further studied.

### Rice flowering enhances the liver metabolism profiles of tilapia in rice-fish symbiosis

The metabolomic provided the comprehensive metabolic profile of biological samples and detailed metabolic responses of the host to different stimuli and environments [33–36]. Our results indicated that compared with the aRT and bTT groups, there were 1015 and 2183 upregulated metabolites in the bRT group, respectively. Moreover, many metabolic pathways including the ABC transporters, choline, pyrimidine, and glycerophospholipid metabolism were upregulated in the bRT group, indicating that rice flowering changed the tilapia metabolic profiles and enhanced the metabolism capacity in rice-fish symbiosis, which was also agreed with the reports that the metabolic pathways and metabolites in fish can be altered by different culture environments [37].

### Rice flowering modulates the microbiota abundance involved in amino acid and lipid metabolism

Many studies have shown that the correlation analysis of 16S sequencing and metabolomics was highly favorable to study the potential mechanisms underlying diseases or treatments [38, 39]. Our analyses showed that compared with the other three groups, the relative abundance of *Bacteroides* was higher, and those of *Pseudomona*, *Cetobacterium*, and *Clostridium* were lower in the bRT group. Spearman’s correlation analysis indicated *Bacteroides* showed a positive correlation with many metabolites which related to amino acid, fatty acid, and lipid metabolism, while *Pseudomona*, *Cetobacterium*, and *Clostridium* showed negative correlations with these relevant metabolites, which were consistent with studies that *Pseudomonas* [40], *Clostridium* [41], and *Cetobacterium* [42] and were all involved in host metabolites and compound anabolism. Therefore, we speculated that rice flowering increases the intestinal microbiota abundance involved in amino acid, fatty acid, and lipid metabolism, resulting in improving the muscle nutrient and quality of tilapia in rice-fish symbiosis through the host microbial-metabolic-phenotype axis, which had been discussed in some studies [43, 44]. However, further investigation is necessary to elucidate their underlying mechanisms.

## Conclusion

In summary, rice flowering improves the tilapia muscle nutrient, intestinal microbiota diversity, and disease resistance and modulates the host metabolism to acclimatize the comprehensive environment in rice-fish symbiosis. Specifically, rice flowering alters the microbiota abundance involved in amino acid, fatty acid, and lipid metabolism, resulting in improving the muscle nutrient and quality through the crosstalk of gut microbial and host metabolism. Our study will not only provide new insights into the gut microbiota-metabolism-phenotype axis, but also strong support for the promotion and application of rice-fish symbiosis in aquaculture.

## Data Availability

The datasets supporting the results and conclusions of this article were deposited in the NCBI Sequence Read Archive database under the accession number PRJNA892587 (microbiota raw sequencing data). All other data are contained within the main manuscript.
